# Dyadobacter helix sp. nov. and Dyadobacter linearis sp. nov., from drinking water

**DOI:** 10.1099/ijsem.0.006570

**Published:** 2024-11-18

**Authors:** Teresa Lucena, María J. Pujalte, David R. Arahal

**Affiliations:** 1 Departamento de Microbiología y Ecología, Universitat de València, Spain

**Keywords:** drinking water, *Dyadobacter*, ecological distribution, functional genomics, phylogenomic analysis, *Spirosomataceae*

## Abstract

The purpose of this study is to better characterize and complete the classification of two bacterial strains, CECT 9275^T^ and CECT 9623^T^, isolated from drinking water systems and affiliated to the genus *Dyadobacter* by partial 16S rRNA gene sequence comparison. Hence, we report here the phenotypic, genomic and phylogenetic characterization performed on these strains. Both strains grow on R2A agar forming mucous, bright yellow colonies, developing at 26 °C in 48 h. They produce flexirubin and are oxidase and catalase positive, mesophilic and non-halophilic. The cells of strain CECT 9275^T^ are curved rods mainly associated in pairs, forming nearly closed rings or resembling the shape of the number three, to long spirals resembling a corkscrew. Its draft genome has an estimated size of 7.23 Mbp (G+C content 45.4%). Strain CECT 9623^T^ appeared on wet mounts as straight rods, mostly in pairs, sometimes forming long filaments (up to 20 µm). Its draft genome is shorter, with an estimated size of 6.45 Mbp (G+C content is 46.1%). Overall genome relatedness indexes clearly define them as separate organisms, so based on all the data collected, we propose the species *Dyadobacter helix* sp. nov. with type strain AB1^T^ (=CECT 9275^T^=LMG 32341^T^) and *Dyadobacter linearis* sp. nov. with type strain AB67^T^ (=CECT 9623^T^=LMG 32342^T^).

## Introduction

The bacterial genus *Dyadobacter* was established by Chelius and Triplett in 2000 [1] to accommodate the species *D. fermentans* isolated from the plant *Zea mays* and has since expanded to reach currently 28 validly named species according to the List of Prokaryotic Names with Standing in Nomenclature (accessed on July 2024) [2]. The genus was considered generalist, in terms of ecological interplay, in a book chapter written when it contained only six species [3], but the same pattern is confirmed now: its species originate from disparate habitats such as a wide variety of soils, plant-associated habitats, sediments, fresh water, seawater and even human-polluted environments [4,13]. The genus is classified in the family *Spirosomataceae*, order *Cytophagales*, class *Cytophagia* and phylum *Bacteroidota*. They are non-motile Gram-negative rods occurring in pairs, aerobic and chemoheterotrophic, producing flexirubin-type yellow pigments and possessing catalase and cytochrome oxidase activities. The genus description has been emended twice [4,14], but none of its species have been reclassified nor transferred from another genus. *Dyadobacter* genomes are notoriously large, attaining sizes of more than 8 Mb in some species, and their G+C molar content is 40–52%.

Two bacterial strains, AB1^T^ and AB67^T^, isolated from drinking water systems [15] and deposited in the Spanish Type Culture Collection [Colección Española de Cultivos Tipo (CECT), Valencia, Spain] as CECT 9275^T^ and CECT 9623^T^, respectively, were affiliated to *Dyadobacter* by 16S rRNA gene sequence comparisons but displayed similarities lower than 98% to any type strain, which is a good indication of taxonomic novelty [16]. Here, we report the phenotypic, genomic and phylogenetic characterization performed on these strains, which led us to confirm their taxonomic novelty, and hence, we describe two new species of *Dyadobacter* each based on one of them.

## Isolation and maintenance

The two strains of *Dyadobacter*, AB1^T^ (=CECT 9275^T^) and AB67^T^ (=CECT 9623^T^), characterized in this study were obtained from the CECT, Spain. According to the records, strain AB1^T^ was isolated from a drinking water treatment plant in Barcelona (Spain) (coordinates 41° 21′ 09.5″ N 2° 02′ 56.2″ E) on 1 August 2016, while strain AB67^T^ was isolated from the drinking water distribution network in Tona (Barcelona, Spain) (41° 50′ 58.7″ N 2° 13′ 45.6″ E) on 23 February 2018. The strains were obtained from the membrane filters incubated at 22 °C on R2A medium for 2 days and Water ISO Plate Count Agar for 3 days, respectively, then streaked on the same media until pure cultures were obtained, submitted to matrix-assisted laser desorption/ionization-time of flight MS bacterial identification procedure and reported as non-identified [15]. The same study allowed to identify them as the only two *Dyadobacter* sp. among a collection of 3809 isolates from several drinking water sources [15]. After deposition in CECT, as CECT 9275^T^ and CECT 9623^T^, respectively, they were further stored in lyophilized format for long-term maintenance, using *m*-inositol (5%) as cryoprotectant. In addition, they were deposited at Laboratorium voor Microbiologie, Universiteit Gent (LMG), Belgium, where they are kept as LMG 32341^T^ and LMG 32342^T^, respectively. Both strains were routinely cultured on R2A medium at 26 °C.

## 16S rRNA gene sequencing and phylogeny

**Fig. 1.**
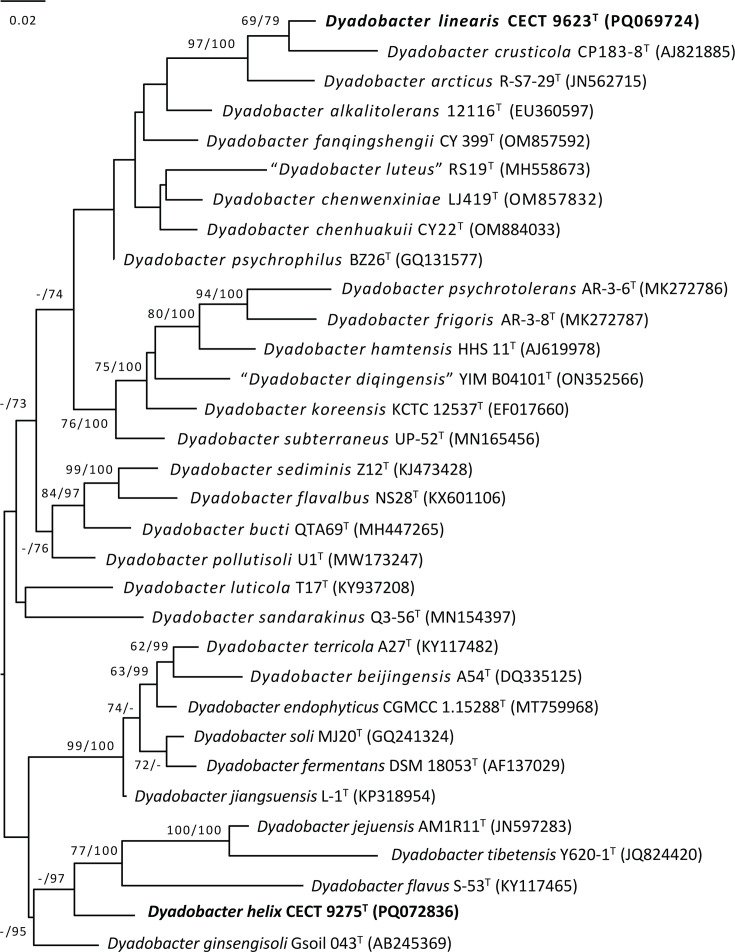
Maximum likelihood (ML) tree inferred from 16S rRNA gene sequences under the GTR+GAMMA model and rooted by midpoint rooting. The branches are scaled in terms of the expected number of substitutions per site. Phylogenies were inferred by the GGDC web server [20] available at http://ggdc.dsmz.de/ using the DSMZ phylogenomic pipeline [21] adapted to single genes. The numbers above the branches are support values when larger than 60% from ML (left) and MP (right) bootstrapping.

A partial 16S rRNA sequence was obtained for each strain by PCR amplification and Sanger sequencing [17] and subsequently compared by basic local alignment search tool (blast) searches [18] that placed the strains as unnamed *Dyadobacter* spp. The complete sequences of 16S rRNA genes were later obtained from the sequenced genomes, and after confirming they matched (100% coincidence) the respective partial sequences, they were compared through blast [18] and EzBiocloud [19] tools to obtain their closest neighbours. A maximum likelihood tree (Fig. 1) was inferred from 16S rRNA gene sequences by the Genome-to-Genome Distance Calculator (GGDC) web server [20] available at http://ggdc.dsmz.de/ using the DSMZ-Deutsche Sammlung von Mikroorganismen und Zellkulturen phylogenomic pipeline [21] adapted to single genes.

The top similarities between the 16S rRNA gene sequence of strain CECT 9275^T^ in blast (with filter type material activated) are 96.8% to *Dyadobacter bucti*, 96.7% to *Dyadobacter ginsengisoli* and 96.6% to *Dyadobacter jiangsuensis* and *Dyadobacter flavalbus*. Regarding strain CECT 9623^T^, these are 98.0% to *Dyadobacter crusticola*, 97.6% to *Dyadobacter arcticus* and 97.1% to *Dyadobacter luteus*.

The position of the novel strains within the *Dyadobacter* phylogenetic tree, as defined on the basis of the 16S rRNA gene, is shown in Fig. 1. While strain CECT 9275^T^ forms a separate branch without a close link to any species, strain CECT 9623^T^ merges with *D. crusticola* and *D. arcticus*.

## Genome features and phylogenomics

Genomic DNA was isolated using Bacteria DNA Preparation Kit (Jena Bioscience) following the standard protocol recommended by the manufacturer. The integrity of the extracted DNA was checked by visualization in a 2.0% (w/v) agarose gel electrophoresis. Its purity and quantity were checked by measuring the absorbance at 260 and 280 nm with a spectrophotometer NanoDrop 2000c (Thermo Scientific) and calculating the ratio A260/A280. The genome sequencing of the strains was achieved at Servicio Central de Soporte a la Investigación Experimental (SCSIE) of the University of Valencia (Valencia, Spain). In the case of strain CECT 9275^T^, this was done using a Sequel PacBio RS II technology (SMRT Link v7.0) and assembled with Hierarchical Genome Assembly Process (HGAP4) *de novo* assembly analysis application. In the case of strain CECT 9623^T^, the genome sequencing was achieved using an Illumina MiSeq technology with 2×250 paired-end reads. The reads were analysed for quality control using FastQC, a common quality control tool developed by Babraham Bioinformatics to check raw sequencing data. After filtering, the remaining reads were assembled using SPAdes 3.14.1 software [22]. A plot, coverage versus length of the contigs, was performed to help in the choice of the parameters for contig filtering. After the filtration of contigs (500 bp length and 10–50×kmer coverage), the evaluation of the final assembly against a reference genome was done with the software QUAST v4.3 [23].

The bioinformatic tool CheckM v1.0.7 [24] was used to assess the genome quality prior to annotation using Prokka v1.12 [25] and Rapid Annotation using Subsystem Technology (RAST) v2.0 [26]. The process of quality assessment of reads, read processing, assembly and annotation with Prokka was carried out in Linux OS; other tools were accessed online. The updated minimal standards for the use of genome data and how they can be applied for taxonomic purposes [16] have been observed in this study.

The similarity between genomes was established using *in silico* DNA–DNA hybridization (isDDH) with the GGDC 2.1 [20], average nucleotide identity with blast (ANIb) algorithm [27] and average amino acid identity (AAI) [28]. Phylogenomic analysis was performed with the Up-to-date Bacterial Core Gene ver.1 [29]. This software tool is available for download at EzBioCloud [19] and employs a set of 92 bacterial core genes that are single copy and commonly present in all bacterial genomes.

The main characteristics of the genomes are presented in Table 1. The draft genome of strain CECT 9275^T^ has an estimated size of 7.23 Mbp. It is composed of four contigs with an N50 value of 4 293 963 nts and final assembly coverage of 140×. CheckM results of contamination and completeness were 0.6 and 100%, respectively. The assembly contains 5824 protein coding sequences and 56 RNA genes. The G+C content is 45.4%. Its 16S rRNA gene sequence is complete and 100% identical to the partial sequence previously amplified by Sanger technology. The draft genome of strain CECT 9623^T^ has an estimated size of 6.45 Mbp. It is composed of 27 contigs with an N50 value of 987 763 nts and final assembly coverage of 127×. CheckM results of contamination and completeness were 0.00 and 100%, respectively. The assembly contains 5376 protein coding sequences and 51 RNA genes. The G+C molar content is 46.1%. Its 16S rRNA gene sequence is complete and 100% coincident with the partial sequence previously amplified by Sanger technology.

**Table 1.** Statistics of the *Dyadobacter* sp. genomes analysed in this study

Strain	Size (Mb)	G+C ratio	Contigs	N50 (Mb)	L50	Accession no.
CECT 9275^T^	7.23	45.4	4	4.3	1	GCF_907164905
CECT 9623^T^	6.45	46.1	27	1.0	3	GCF_907165045
*D. alkalitolerans* DSM 23607^T^	6.29	45.7	51	0.4	6	GCF_000428845
*D. arcticus* DSM 102865^T^	6.04	43.9	13	1.3	2	GCF_011762185
*D. beijingensis* DSM 21582^T^	7.38	52.1	16	1.2	3	GCF_000382205
*D. bucti* QTA69^T^	8.41	46.0	39	0.7	4	GCF_005869225
*D. chenhuakuii* CY22^T^	6.08	45.8	2	6.0	1	GCF_023821985
*D. chenwenxiniae* LJ419^T^	7.17	45.2	1	7.2	1	GCF_022869785
*D. crusticola* DSM 16708^T^	6.17	46.7	45	0.5	6	GCF_000701505
*D. diqingensis* YIM B04101^T^	7.27	43.5	28	0.8	4	GCF_024220585
*D. endophyticus* CGMCC1.15288^T^	7.90	49.6	30	1.8	2	GCF_014641595
*D. fanqingshengii* CY399^T^	6.27	45.2	2	6.3	1	GCF_023822005
*D. fermentans* DSM 18053^T^	6.97	51.5	1	7.0	1	GCF_000023125
*D. flavalbus* NS28^T^	6.48	44.8	79	0.3	6	GCF_006149045
*D. frigoris* AR-3-8^T^	8.33	40.1	67	0.4	8	GCF_005280585
*D. jejuensis* DSM 100346^T^	5.76	45.4	73	0.3	6	GCF_003149085
*D. jiangsuensis* DSM 29057^T^	8.27	50.3	48	0.4	7	GCF_003014695
*D. koreensis* DSM 19938^T^	7.34	41.3	26	0.5	5	GCF_900108855
*D. luteus* RS19^T^	6.95	43.0	59	0.2	10	GCF_003383615
*D. luticola* T17^T^	6.41	46.9	16	2.0	2	GCF_005860805
*D. pollutisoli* U1^T^	7.69	45.5	1	7.7	1	GCF_026625565
*D. psychrophilus* DSM 22270^T^	6.74	45.1	34	0.5	3	GCF_900167945
*D. psychrotolerans* AR-3-6^T^	7.93	42.1	44	0.7	5	GCF_004349265
*D. sandarakinus* Q3-56^T^	6.07	49.1	1	6.1	1	GCF_016894445
*D. sediminis* Z12^T^	6.20	44.9	35	1.2	3	GCF_005860765
*D. soli* DSM 25329^T^	8.75	50.5	47	0.4	10	GCF_900101885
*D. subterraneus* UP-52^T^	7.79	40.0	21	6.1	1	GCF_015221875
*D. tibetensis* Y620-1^T^	5.31	43.4	33	0.8	3	GCF_000566685

Table 2 shows the values obtained for the three overall genomic relatedness indexes that were explored, namely, ANIb, isDDH and AAI, relating the genomes of the novel strains with those of the type strains of neighbouring, validly named species and references used for comparison. The most remarkable finding for the purpose of taxonomic classification is that both strains show ANIb and isDDH values lower than the respective species thresholds (ANIb 95% and isDDH 70%), thus qualifying each of them as different genomic species.

**Table 2.** Overall genome relatedness indexes between strains CECT 9275^T^ and CECT 9623^T^ (in bold) to type strains of *Dyadobacter* species

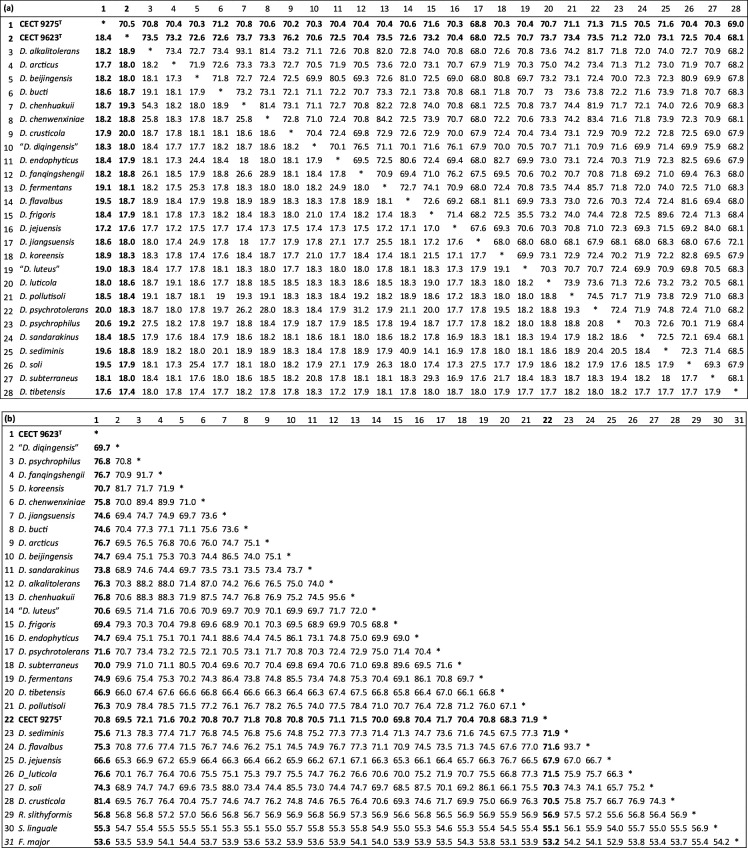 ­

(a) ANIb (top right) and isDDH (bottom left); (b) AAI.

AAI index, also useful to explore genus-level relatedness, ranks from 67 to 81% when comparing strain CECT 9623^T^ to other *Dyadobacter* species, being the highest value with *D. crusticola*. AAI values to species in other genera are 54–57%. Slightly lower values are found between strain CECT 9275^T^ and other *Dyadobacter* species (68–72%) and to species on neighbour genera, *Flectobacillus*, *Spirosoma* and *Runella* (53–57%).

**Fig. 2.**
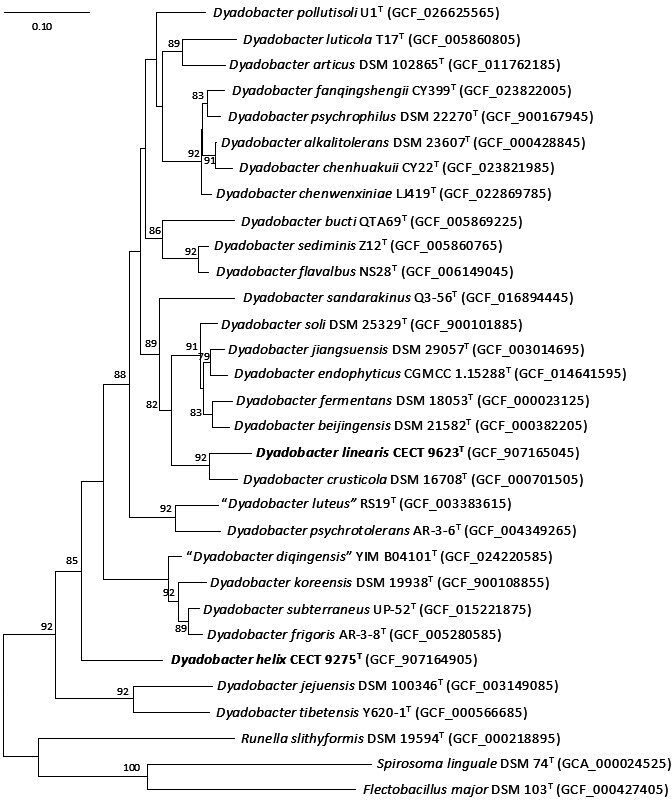
Phylogenetic tree generated with the Up-to-date Bacterial Core Gene ver.1 [29] by using whole-genome aa sequences. The numbers at the nodes indicate the gene support index (maximal value is 92). Genome accession numbers are indicated in parentheses. Bar, 0.05 substitutions per position.

The phylogenomic tree, based on 92 core genes, is shown in Fig. 2. It confirms the close phylogenetic relationship of CECT 9623^T^ to *D. crusticola*, with very high node support, in agreement with the data on 16S rRNA gene similarity, ANIb, isDDH and AAI relatedness levels.

## Functional genomics

The genomes of the two strains, annotated through RAST [26] and Prokka [25], were examined directly. One of the most notable characteristics of their gene content was the large amount of varied CAZyme genes, including several genes encoding for polysaccharide-related enzymatic activities (Table S1, available in the online version of this article).

Among the gene characteristics of the phylum *Bacteroidota*, the most prominent was the pair formed by *susC* and *susD* genes for the components of the PUL system of *Bacteroides* [‘Outer membrane TonB-dependent transporter, utilization system for glycans and polysaccharides (PUL), SusC family’ and ‘Cell surface glycan-binding lipoprotein, utilization system for glycans and polysaccharides (PUL), SusD family’]. This pair of genes, usually (but not always) accompanied by genes for anti-FecI sigma factor FecR and for an RNApol ECF-type sigma factor, is present in 96 copies in the genome of strain CECT 9275^T^ and 81 copies in the genome of strain CECT 9623^T^, according to RAST annotations. Also, many copies of a gene identified as ‘acidobacterial duplicated orphan permease of unknown function’ in RAST annotations are present in both genomes (28 and 33 copies, respectively).

Several *gld* genes are present (*gldA*, *B*, *C*, *D*, *F*, *G*, *H*, *J* and *N*), as it is usual in the members of this phylum, but a gene coding for the gliding-related adhesin SprB is missing, in agreement with the non-motile behaviour of the cells. They also contain the genes coding for the *Bacteroides*-aerotolerance proteins BatA, B and C. Both strains contain a single carotenoid synthetic operon, under the control of a transcription regulator *merR*. The operon includes transcriptional regulator, *merR* family, phytoene dehydrogenase (EC 1.14.99.-), phytoene synthase (EC 2.5.1.32), isopentenyl-diphosphate δ-isomerase (EC 5.3.3.2), β-carotene hydroxylase and lycopene cyclase. This cluster matches the one described for *Dyadobacter* sp. 32 as a putative zeaxanthin biosynthetic gene cluster [30]. Other common genes are the ones encoding the toxin–antitoxin system HigA–HigB.

Both strains present the heptaprenyl diphosphate synthase gene, suggesting that menaquinone-7 should be the major menaquinone, and the phosphatidylserine decarboxylase gene, suggesting the presence of phosphatidylethanolamine as the major membrane polar lipid. This is coincident with the chemotaxonomic signatures of the genus *Dyadobacter*. Other interesting genes for metabolic activities shared by the two strains are the ones encoding for phospholipase C, sphingomyelinase, carbonic anhydrase, phytase, polyphosphate kinase, exopolyphosphatase and some mono- and dioxygenases (including catechol-2,3-dioxygenase). Transcriptional regulators of the AraC and Lux R families are also abundant.

It is noteworthy the presence of several transposase genes (66 in strain CECT 9275^T^ and 8 in CECT 9623^T^) and a type I restriction–modification system. CRISPR arrays are absent in both strains, but CECT 9275^T^ contains Cas1 and Cas2 genes. Genes exclusively present in CECT 9275^T^ comprise a nitrous oxide reductase and a conjugative transposon with several Tra genes. Conversely, genes unique to CECT 9623^T^ are the cyanophycinase, cyanophycin synthase and isoaspartyl aminopeptidase genes, allowing the strain the potential synthesis and degradation of cyanophycin. It also contains genes for the circadian clock proteins KaiB and KaiC, which are common in *Cyanobacteriota* but rarely found among *Bacteroidota* [31].

## Phenotypic characterization

Colonial morphology and pigmentation were registered after 48–72-h incubation. The KOH test for flexirubin-type pigment was done according to Bernardet *et al*. [32]. Gram-reaction tests were performed following the method described in [33]. The cell morphology, size and motility were observed on wet mounts by phase-contrast microscopy in a Leica DMRB fluorescence microscopy. The cell morphology of strain CECT 9275^T^ was also determined by using the scanning electron microscopy (SEM) at the SCSIE (University of Valencia). For these observations, the cells were filtered with a 47-mm polycarbonate membrane filter of 0.2-µm pore size (Millipore) with a peristaltic pump. Filters were fixed in glutaraldehyde with a final concentration of 3% at room temperature and washed three times with 0.1 M phosphate buffer. A series of sequential ethanol dehydrations were performed for 10 min each (50, 70, 95 and 100 %) and post-fixed with 2% osmium before drying the samples under CO_2_ using a critical point drier apparatus (CPD030, Baltec). The samples were examined in a Hitachi S4800 field emission scanning microscope with field emission gun and a resolution of 1.4 nm to 1 kV. Pictures were stored digitally and processed using the software Quantax 400.

Temperature, pH and salinity ranges for growth were determined on R2A plates (temperatures: 4, 15, 28, 30, 37 and 40 °C), R2A broth adjusted to different pHs (4.0–10.5, at 0.5 intervals) and R2A broth with added sea salts (0–6%, at 0.5% intervals), respectively. Oxidase and catalase activities, hydrolysis of casein, starch, Tween 80 and DNA were tested as previously reported [34]. Cellulose degradation was tested in R2A broth with submerged strips of Whatman filter paper after 21 days of incubation.

API ZYM, API 20NE and API 50CH/E (bioMérieux) were used to determine the enzymatic and metabolic characteristics and ability to acidify different carbohydrates by the strains. API ZYM strips were incubated 6 h at 26 °C, while API 20NE and API 50CH/E were incubated at the same temperature but recorded at 24 and 48 h.

Screening of sole carbon and energy sources used for growth was performed on the basal medium described by Chaturvedi *et al*. [5]. The medium contains (w/v) K_2_HPO_4_ 1.05%, KH_2_PO_4_ 0.45%, (NH_4_)_2_SO_4_ 0.1% and purified agar (Oxoid) 1.5% in ultrapure water, plus 0.5% of the following carbon sources: d-ribose, l-arabinose, d-xylose, d-fructose, d-galactose, d-mannose, l-rhamnose, l-sorbose, l-melibiose, d-glycerol, d-mannitol, d-sorbitol, m-inositol, acetate, citrate, fumarate, lactate, glycine, l-alanine, l-aspartate, l-glutamate, l-leucine, l-proline, l-serine, l-threonine, l-tyrosine, l-arginine, l-tryptophan and l-histidine. Yeast extract was used as a positive control, and medium without any carbon source added was used as a negative control. Plates were inoculated using a multipoint inoculator from cultures in R2A broth.

**Fig. 3.**
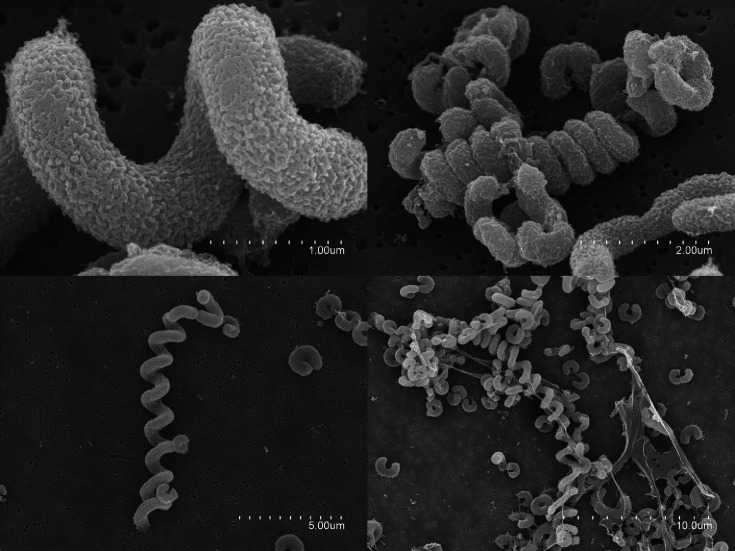
Cellular morphology of strain CECT 9275^T^ as seen under SEM at different scales to show differences in length among cells whose morphology ranges from C shaped to long spirals.

Microscopic observations revealed that the cells of strain CECT 9275^T^ are curved rods mainly associated in pairs, forming nearly closed rings or resembling the shape of the number three. Additionally, long spirals resembling a corkscrew are also seen. The SEM reveals the spirals to be continuous, with division rendering individual cells and pairs of cells at the end of the spirals. Spiral length is around 12 µm (Fig. 3). The cells, pairs and spirals were partially covered with extracellular adherent material. It is noticeable that spiral morphology is not explicitly included in the description of the genus *Dyadobacter* [1], although it mentions the possibility of curved arrangements, in addition to straight rods, which is the morphology of strain CECT 9623^T^ on wet mounts, mostly in pairs, sometimes forming long filaments (up to 20 µm).

Both strains grow on R2A agar forming mucous, bright yellow colonies, developing at 26 °C in 48 h. They do not grow on Luria-Bertani agar. Flexirubin reaction test was positive, indicating that the two strains produce this type of pigments rather than carotenoids. Other common traits were the positive response to oxidase and catalase, their mesophilic and non-halophilic character and a general lack of reactivity on carbohydrate acidification tests, hydrolysis of polymers and use of sole carbon sources (probably due to some nutritional requirement in the case of CECT 9275^T^, as it was able to grow on control medium with yeast extract). In contrast, enzymatic activities determined in API ZYM strips were diverse. Detailed results for each strain are listed in the corresponding species descriptions and in Table 3.

**Table 3.** Differential phenotypic characteristics between strains CECT 9275^T^ and CECT 9623^T^ compared to several related *Dyadobacter* species

Trait	1	2	3	4	5	6	7	8	9	10	11	12
Helical morphology	+	−	−	−	−	−	−	−	−	−	−	−
Oxidase	+	+	+	+	+	+	+	−	+	+	+	nd
Upper growth temperature (°C)	37	37	37	30	28	30	30	35	30	35	37	35
Growth at 4 °C	−	−	−	+	+	−		+	+	−	−	+
Maximum pH	10	10	8	8	9	8	10	9	8.5	11	10	nd
Maximum NaCl (%)	1.5	1.5	1.5	1	1	nd	1.5	2	1	1.5	3	nd
Nitrate reduction to nitrite	−	−	−	−	−	−	−	+	+	−	−	nd
Urease	−	−	−	−	−	−	+	+	−	+	−	nd
Gelatin hydrolysis	+	−	−	−	−	+	+	−	−	+	−	nd
Starch hydrolysis	−	−	−	−	−	+	+	+	−	+	−	+
Arabinose assimilation	−	−	+	−	−	+	+	−	+	−	−	+
API ZYM												
Esterase	−	−	+	+	+	+	nd	+	−	nd	nd	+
Esterase lipase	−	−	+	+	+	−	nd	−	nd	nd	nd	+
Cystine arylamidase	−	−	+	−	−	+	−	+	−	nd	+	+
Trypsin	−	−	+	+	−	−	nd	−	−	nd	+	−

1, CECT 9275T; 2, CECT 9623T; 3, *D. fermentans* NS114T [1]; 4, *D. crusticola* CP183-8T [4]; 5, *D. arcticus* R-S7-29T [6]; 6, *D. soli* MJ20T [7]; 7, *D. jiangsuensis* L-1T [8]; 8, *D. bucti* QTA69T [9]; 9, *D. ginsengisoli* Gsoil043T [10]; 10, *D. flavalbus* NS28T [11]; 11, *D. jejuensis* AM1R11T [12]; 12, *D. tibetensis* Y620-1T [13]. +, positive; –, negative; nd, no data available.

The major cellular fatty acid composition was determined by following the previously described procedures [34]. Strain CECT 9275^T^ was cultivated on R2A at 26 °C for 6 days, while CECT 9623^T^ was cultivated in the same medium and temperature for 3 days. The biomass was prepared according to standard protocols as described for the MIDI Microbial Identification System [35]. The cellular fatty acid content was analysed by gas chromatography with an Agilent 6850 chromatographic unit, with the MIDI Microbial Identification System using the TSBA6 method [36] and identified using the Microbial Identification Sherlock software package. The major fatty acids detected were iso C_15:0_ and summed feature 3 (C_16:1_* ω7*c*/*C1_6:1_* ω6*c), with minor amounts of other components (Table S2), in general agreement with the composition reported for *Dyadobacter* [1,4].

## Ecology

To assess the possible ecological distribution of the novel strains, a similarity search was performed using different strategies. Starting with 16S rRNA sequences, unfiltered blast searches yielded a rather moderate top identity in the case of strain CECT 9275^T^, namely, 97.82% to sequence HQ120850 corresponding to an uncultured bacterium from a loamy sand collected from a field planted with tomatoes at California (USA). The online tool MAPseq [37] showed a preference for animal samples (2781) followed by unknown sources (2455), soil (517), plant (312) and aquatic samples (233) working at a 99% OTU level, but the confidence was 0. The confidence increased to 0.5974 if the OUT level was reduced to 90%. This also has the effect of increasing the relative abundance of aquatic samples from 3.7 to 10% but still accounting very modestly as the natural niche of this organism. Finally, Protologger [38] did not identify any metagenome-assembled genome (MAG) matching our query, an indication of novelty, and pointed to the following amplicon samples as the most common sources for detection: rhizosphere (14.7%), wastewater (11.6%) and activated sludge (11.3%) but in all cases with very low mean relative abundances (maximum 0.08% in activated sludge metagenome). We can conclude that it has a relatively wide and diverse distribution but very low abundance.

As for strain CECT 9623^T^, the top similarity in the unfiltered blast search was 99.2% to sequence OM856830 whose source is not detailed. According to MAPseq [37] and applying the 99% OTU level, the sample distribution was animal (2335), unknown (810), plant (647), soil (267) and aquatic (102) with a 0.5399 confidence. Protologger [38] did not identify any MAG matching this genome and pointed to the following amplicon samples as the most common sources for detection: rhizosphere (48.0%), soil (18.4%) and plant (16.9%) but in all cases with low mean relative abundances (maximum 0.34% in insect gut metagenome). We can conclude that it has a very wide and diverse distribution but low abundance.

## Discussion and taxonomic conclusions

This study provides sufficient data to propose a sound classification of strains CECT 9275^T^ and CECT 9623^T^ as novel species of the genus *Dyadobacter*. Genome relatedness to any other type strain is below the generally accepted cut-off values [16], and the coherence of the recipient genus, both phylogenetically and according to AAI, has been assessed. At the same time, both strains have been thoroughly described and compared to their closest relatives to highlight differential traits, and they have also been analysed for their functional potential and ecological distribution. These data permit us to formally propose the species *Dyadobacter helix* sp. nov. and *Dyadobacter linearis* sp. nov.

## Description of *Dyadobacter helix* sp. nov.

*Dyadobacter helix* (he’lix. L. fem. n. *helix*, a whorl).

The cells are Gram-reaction-negative, curved rods (0.5×1.0 µm) in pairs and helices (around 12 µm long). Non motile. Colonies in R2A medium are mucous and yellow pigmented. Flexirubin pigment reaction to KOH is positive. Oxidase and catalase positive. Strictly aerobic, not able to ferment carbohydrates, not able to reduce nitrate to nitrite or N_2_. Mesophilic, growth is positive from 15 to 37 °C but not at 4 or 40 °C. Grows at 0% salinity and up to 1.5% but not at 3% or more. pH range is 5.0 to 10.0. Unable to hydrolyse casein, starch, cellulose, Tween 80 or DNA, but proteolytic on gelatin. Enzymatic activities in API ZYM are positive for alkaline and acid phosphatases, leucine arylamidase, valine arylamidase (weak), naphthol-AS-BI-phosphohydrolase, α-galactosidase (weak), β-galactosidase, α- and β-glucosidases and *N*-acetyl-β-glucosaminidase, while esterase, esterase lipase, lipase, cystine arylamidase, trypsin, α-chymotrypsin, β-glucuronidase, α-mannosidase and α-fucosidase are negative. Positive for aesculin and gelatin hydrolysis and p-nitrophenyl α-d-glucopyranoside test (β-galactosidase) and assimilation of *N*-acetyl glucosamine, glucose, mannose and maltose in API 20NE, but negative for nitrate reduction, glucose fermentation, indole production from tryptophan, arginine dihydrolase and urease. Weak acidification of galactose, glucose, fructose, *N*-acetyl-d-glucosamine, amygdalin, arbutin, salicin, cellobiose, maltose, lactose, turanose and lyxose in aerobic API 50CH/E. Growth in the minimal medium is positive only with yeast extract (positive control) and with l-tryptophan, out of 29 sole carbon sources tested. The main cellular fatty acids are iso C_15:0_ and summed feature 3 (C_16:1_* ω7c/*C1_6:1_ ω6c). G+C content of its DNA is 45.4%.

The type strain is AB1^T^ (=CECT 9275^T^=LMG 32341^T^), isolated from a drinking water treatment plant in Barcelona (Spain). The GenBank/EMBL/DDBJ accession numbers for the 16S rRNA gene and genome sequences of the type strain are PQ072836 and CAJRAF01, respectively.

## Description of *Dyadobacter linearis* sp. nov.

*Dyadobacter linearis* (li.ne.a’ris. L. masc. adj. *linearis*, linear).

The cells are rods (0.5–1.0×1.0–3.0 µm), Gram-reaction-negative, mostly in pairs or rarely in linear chains. Non motile. Colonies on R2A medium are mucous and bright yellow. Pigment is flexirubin type, according to the KOH test. Oxidase and catalase positive. Strictly aerobic. Mesophilic, neutrophilic and non-halophilic, growing in the following ranges: 15–37 °C, 5.5 to 10 pH and from 0 to 1.5% NaCl. Nitrate reduction to nitrite or gas is negative. Glucose is not fermented. Negative for hydrolysis of starch, cellulose, casein, gelatin, Tween 80 and DNA. Negative for urease, indole production from tryptophan and arginine dihydrolase. Positive for β-galactosidase, aesculin hydrolysis and delayed assimilation of glucose, maltose, mannitol and *N*-acetyl-d-glucosamine (API 20NE). Enzymatic activities detected in API ZYM include alkaline phosphatase, leucine and valine arylamidases, α- and β-galactosidases, α- and β-glucosidases and *N*-acetyl-β-glucosaminidase. A weak response is obtained for acid phosphatase, naphthol-AS-BI-phosphohydrolase and α-mannosidase. Esterase, esterase lipase, lipase, cystine arylamidase, trypsin, α-chymotrypsin, β-glucuronidase and α-fucosidase are negative. Weak acidification of the following carbohydrates is observed in API 50CH/E strips incubated aerobically: glucose, methyl α-d-mannopyranoside, methyl α-d-glucopyranoside, *N*-acetyl d-glucosamine, amygdalin, arbutin, salicin, cellobiose, maltose, lactose and sucrose, in addition to a strong positive reaction in aesculin. Unable to grow on any of the 30 sole carbon sources assayed in minimal medium, including yeast extract, suggesting that it has non-identified growth factor requirements.

The type strain is AB67^T^ (=CECT 9623^T^=LMG 32342^T^), isolated from a drinking water distribution network in Tona (Barcelona, Spain). The GenBank/EMBL/DDBJ accession numbers for the 16S rRNA gene and genome sequences of the type strain are PQ069724 and CAJRAU01, respectively.

## supplementary material

10.1099/ijsem.0.006570 Uncited Supplementary Material 1.

## Abbreviations

AAI: average amino acid identity
ANIb: average nucleotide identity with BLAST
BLAST: basic local alignment search tool
CECT: Colección Española de Cultivos Tipo
GGDC: Genome-to-Genome Distance Calculator
isDDH: in silico DNA–DNA hybridization
LMG: Laboratorium voor Microbiologie, Universiteit Gent
RAST: Rapid Annotation using Subsystem Technology
SCSIE: Servicio Central de Soporte a la Investigación Experimental (University of Valencia)
