# Supplementary material for: Dyadobacter helix sp. nov. and Dyadobacter linearis sp. nov., from drinking water
Source: Int J Syst Evol Microbiol. 2024 Nov 18;74(11):006570. doi: 10.1099/ijsem.0.006570 (PMC11573002; doi:10.1099/ijsem.0.006570)
Supplement: Uncited Supplementary Material 1. [file ijsem-74-06570-s001.pdf]

Supplementary Material

Dyadobacter helix sp. nov. and *Dyadobacter linearis* sp. nov., from drinking water

Teresa Lucena, María J. Pujalte and David R. Arahal*

Departamento de Microbiología y Ecología, Universitat de València, Spain

Corresponding author

David R. Arahal arahal@uv.es

Table S1. Polysaccharide related enzymes predicted from the genomes (Prokka annotation) of 1, *Dyadobacter helix* CECT 9275^T; 2, *Dyadobacter linearis* CECT 9623^T. Numbers indicate copy number of the corresponding genes.

**Activity**	**1**	**2**
Acetylxylan esterase	3	2
Alginate biosynthesis protein AlgA	1	1
α-Amylase	2	1
Chitinase A1	2	1
Endoglucanase C307	1	1
Endo-1,4-β-xylanase/feruloyl esterase	0	1
Endo-1,4-β-xylanase B	1	1
Endo-1,4-β-xylanase Z (2)	0	2
Extracellular endo-α-(1→5)-L-arabinanase 1	1	0
Extracellular exo-α-(1→5)-L-arabinofuranosidase	0	1
Extracellular xylan exo-α-(1→2)-glucuronosidase	1	1
1,4-α-Glucan branching enzyme GlgB	2	3
1,4-β-D-Glucan glucohydrolase	1	1
α-1,4-Glucan:maltose-1-phosphate maltosyltransferase 1	1	1
β-Glucanase	1	2
Levanase	3	1
Mannan endo-1,4-β-mannosidase (3)	0	3
β-Mannanase/endoglucanase A	0	1
Xyloglucan-specific endo-β-1,4-glucanase BoGH9A	0	1

Table S2. Fatty acid composition of 1, *Dyadobacter helix* CECT 9275^T; 2, *Dyadobacter linearis* CECT 9623^T. All data from this study. -, not detected. tr, traces (less than 1 %). Major fatty acids (> 10 %) are highlighted in bold.

**Fatty acid (%)**	**1**	**2**
Hydroxylated		
iso $C_{15:0}$ 3OH	4.9	2.8
iso $C_{16:0}$ 3OH	1.0	tr
iso $C_{17:0}$ 3OH	8.0	**12.2**
$C_{16:0}$ 3OH	2.9	1.9
Branched		
**iso $C_{15:0}$**	**27.4**	**18.8**
anteiso $C_{15:0}$	1.0	tr
Saturated		
$C_{10:0}$	-	1.7
$C_{14:0}$	1.5	tr
$C_{16:0}$	6.6	4.7
Unsaturated		
$C_{15:1}$ $\omega 5c$	2.6	-
$C_{16:1}$ $\omega 5c$	7.2	9.8
**Summed Feature 3 ($C_{16:1}$ $\omega 7c$/$C_{16:1}$ $\omega 6c$)**	**30.8**	**41.2**
$C_{17:1}$ $\omega 5c$	1.1	-
Summed Feature 9 ($C_{16:0}$ 10-methyl/iso $C_{17:1}$ $\omega 9c$)	tr	1.0
$C_{18:1}$ $\omega 9c$	1.9	2.7
